# Extracellular matrix remodelling in degenerative cervical myelopathy

**DOI:** 10.1093/braincomms/fcag239

**Published:** 2026-07-23

**Authors:** Noah D Poulin, Sydney Brockie, Koby Baranes, James Hong, Cindy Zhou, Sarah Sadat, Mark R Kotter, Michael G Fehlings

**Affiliations:** Department of Clinical Neurosciences, University of Cambridge, Cambridge CB2 0QQ, UK; Welcome-MRC Cambridge Stem Cell Institute, Cambridge CB2 0AW, UK; Division of Genetics and Development, Krembil Research Institute, University Health Network, Toronto, Canada M5T 2S8; Division of Genetics and Development, Krembil Research Institute, University Health Network, Toronto, Canada M5T 2S8; Department of Clinical Neurosciences, University of Cambridge, Cambridge CB2 0QQ, UK; Welcome-MRC Cambridge Stem Cell Institute, Cambridge CB2 0AW, UK; Division of Genetics and Development, Krembil Research Institute, University Health Network, Toronto, Canada M5T 2S8; Division of Genetics and Development, Krembil Research Institute, University Health Network, Toronto, Canada M5T 2S8; Division of Genetics and Development, Krembil Research Institute, University Health Network, Toronto, Canada M5T 2S8; Department of Clinical Neurosciences, University of Cambridge, Cambridge CB2 0QQ, UK; Welcome-MRC Cambridge Stem Cell Institute, Cambridge CB2 0AW, UK; Division of Genetics and Development, Krembil Research Institute, University Health Network, Toronto, Canada M5T 2S8; Division of Neurosurgery and Spine Program, Department of Surgery, University of Toronto, Toronto, Canada M5T 1P5

**Keywords:** blood–spinal cord barrier, matrisome, chronic compression, astrogliosis, fibrosis

## Abstract

Degenerative cervical myelopathy (DCM), a type of spinal cord injury triggered by chronic compression from degenerative changes of the spine, induces pathophysiological changes similar to traumatic spinal cord injury, including reactive gliosis, demyelination and neuron loss. However, the effects of chronic spinal cord compression on extracellular matrix composition and organization remain uncharacterized.

Here, we analyse untreated post-mortem human tissue and a mouse model of chronic spinal cord compression using immunohistochemical and transcriptomic approaches to assess chondroitin sulphate proteoglycan (CSPG) and fibrosis-related matrix deposition.

In human post-mortem tissue, astrogliosis, CSPG accumulation and fibrotic collagen deposition were elevated in the DCM cases (*n* = 7) compared to controls (*n* = 5), though gliosis and fibrosis-related matrix deposition were not significantly associated with the degree of cord compression. The mouse model, however, demonstrated more distinct border-forming astrocyte phenotypes. Transcriptional and histological markers of vascular and interstitial fibrosis were also significantly increased in the mouse model.

These findings demonstrate that astroglial CSPG deposition and fibrotic scarring occur in DCM, albeit potentially in a more diffuse pattern than in traumatic spinal cord injury. Moreover, this study supports previous observations of vascular fibrotic thickening in DCM.

## Teaser

Poulin *et al*. report that degenerative cervical myelopathy is associated with astrogliosis- and fibrosis-related extracellular matrix changes in human tissue and a mouse model, identifying a vascular and interstitial extracellular matrix signature of chronic spinal cord compression distinct from acute spinal cord injury.

## Graphical Abstract

**Graphical Abstract:**
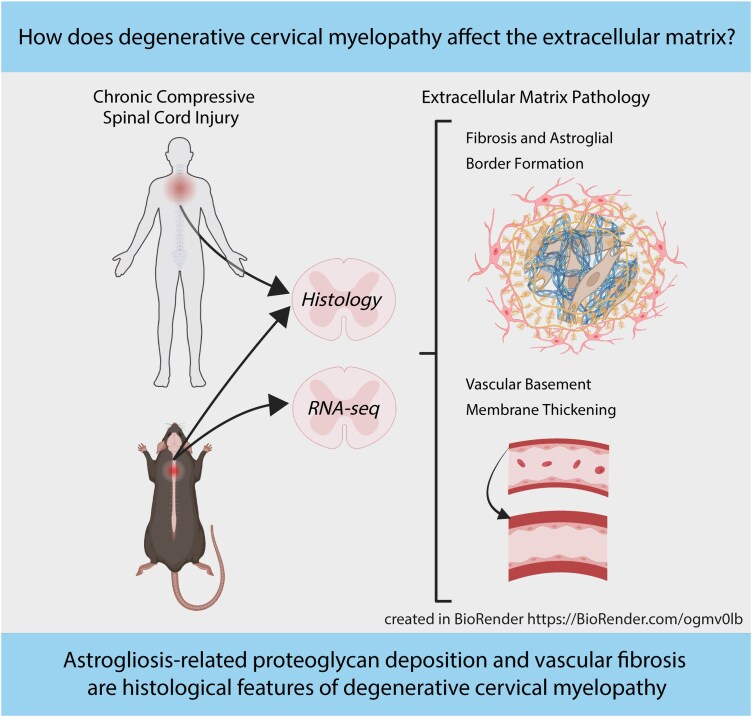

## Introduction

Degenerative cervical myelopathy (DCM) is a leading cause of spinal cord dysfunction in adults.^1^ Over time, mechanical stress, along with age-related vertebral, ligamentous and muscular changes, contributes to spinal degeneration, canal narrowing and chronic spinal cord compression. Common aetiologies of DCM include cervical spondylosis and ossification of the posterior longitudinal ligament (OPLL).^1^ Static pathophysiological factors include intervertebral disc degeneration, ligamentous hypertrophy and congenital canal stenosis.^1-3^ Dynamic factors like transient canal narrowing and hyperextension may further exacerbate compression.^4^

While in most individuals chronic cord compression remains asymptomatic, ∼10% develop DCM.^5^ This has led to the hypothesis that in DCM patients, a vulnerability of the spinal cord exists,^6^ for example, determined by a dysfunction in autophagy^7^ or apolipoprotein E allele^8^ that predisposes to the condition and which may result in a secondary injury cascade that is similar, but likely not identical, to those seen in acute traumatic spinal cord injury (SCI).^9^

In SCI, the secondary injury cascade involves dramatic alterations of the extracellular matrix (ECM). Fibrosis is a recognized prominent feature,^10,11^ and the integrity of the blood–spinal cord barrier (BSCB) is compromised by both mechanical compression and secondary biochemical disruptions. In SCI, astrocytes, oligodendrocyte progenitor cells and pericytes secrete chondroitin sulphate proteoglycans (CSPG) at the lesion border.^12,13^ These inhibit axonal growth, but given appropriate extrinsic and intrinsic conditions, axons may traverse CSPG-rich environments.^14-16^

In contrast, little is known about the ECM in DCM. Although gliosis is a recognized feature of chronic compressive injury in human histological studies,^17-19^ the molecular signature of astrogliosis-related matrix deposition in DCM remains unexplored. Similarly, fibrosis has not been studied extensively in the context of DCM. While, in rodent models of DCM, BSCB disruption is well documented,^20,21^ human histological data on changes to the vascular basement membrane remain scarce. In DCM, elevated CSF albumin and immunoglobulin levels correlate with clinical severity, suggesting some level of BSCB dysfunction in human subjects.^22^ Ischaemia and oxidative stress likely exacerbate vascular permeability,^23,24^ while endothelial damage results from a combination of these factors and mechanical strain. Additionally, neutrophil-derived metalloproteinases contribute to BSCB breakdown in SCI^12,25^ and may play a similar role in DCM.^21^ In post-mortem human DCM tissue, vascular fibrosis—characterized by vessel wall thickening—has been observed in intramedullary vessels,^26-29^ particularly at the region of compression^30^ and in the small veins surrounding cystic necrosis.^31^

This study aimed to describe changes in fibrotic scar-, glial border- and vascular-associated ECM in DCM.

## Materials and methods

### Experimental mouse DCM induction

**Figure 1:**
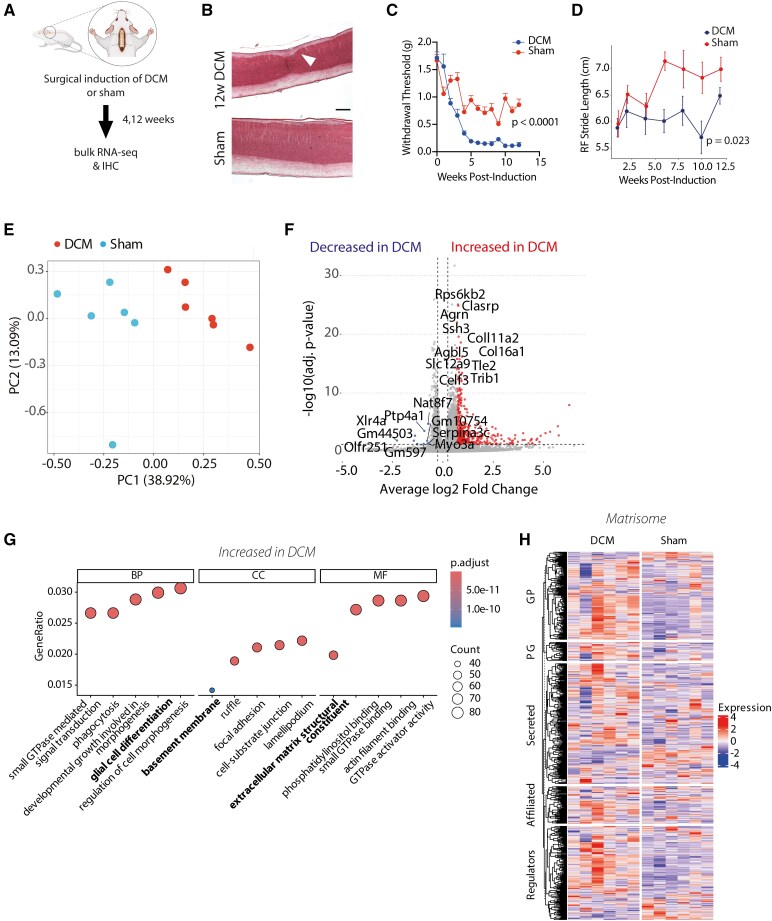
**Transcriptomic signatures of chronic compressive cord injury in a mouse model of DCM.** (**A**) Study design, created in BioRender. https://BioRender.com/i1bh1ms (**B**) Representative haematoxylin and eosin-stained images of the spinal cord compression epicentre (scale bar = 500 um). White arrow denotes area of compression and grey matter loss. (**C**) Von Frey filament testing for mechanical allodynia (two-way ANOVA, *n* = 12 DCM mice, *n* = 12 sham mice). (**D**) Catwalk gait analysis of right forelimb (RF) stride length (*n* = at least 6 mice per group, two-way ANOVA, details in Supplementary Table 3). Data points represent the mean of individual animals ± standard error of the mean. (**E**) Principal components plot of gene expression for DCM and control samples. (**F**) Volcano plot of differential gene expression between DCM and sham animals (*n* = 6 DCM mice, *n* = 6 sham mice). (**G**) GO overrepresentation analysis of genes upregulated in DCM. BP: biological process, MF: molecular function, CC: cellular compartment. (**H**) Heatmap of matrisome gene expression by protein class; columns represent individual animals (*n* = 6).

All experimental procedures were approved by the Krembil Research Institute Animal Resource Center Committee. The 8-week-old male and female C57BL6/J mice underwent surgical implantation of an ossifying polyether material under the C5–6 vertebrae, between the lamina and the spinal cord as previously described^8,32,33^ (Fig. 1A). Anaesthesia was induced using 5% isoflurane then reduced to 2.5% for the duration of the procedure. The surgical area was disinfected using iodine and 70% ethanol. A midline incision was made from the base of the skull to the level of T2. The dorsal aspect of the cervical to mid-thoracic spinal column was exposed using blunt dissection. After removal of the inter-lamina tissue using a surgical hook and scalpel, laminae were lifted away from the cord using a shook and the ossifying material was placed under C5/6. The incision was closed with continuous #5 suture. A 1 ml bolus dose^8,32,33^ of NaCl and buprenorphine (0.05 mg/kg) was administered subcutaneously. Anaesthesia was withdrawn and animals were allowed to recover under a heat lamp. Buprenorphine was administered every 8–12 h for 48 h. Locomotor function measurements were performed in a behavioural cohort of female mice using a Noldus CatWalk system, and mechanical allodynia was evaluated using Von Frey Filament testing. Principles of randomization and blinding were maintained for all animal experiments. Unblinding occurred at the 4-week timepoint to identify DCM animals for sacrifice. No animals were excluded, DCM and sham animals were randomly induced and caged together, and sample sizes were determined based on prior unpublished experiments using this mouse model.

### Post-mortem human tissue histology

Formalin-fixed, paraffin-embedded spinal cord tissues from untreated DCM cases and controls were obtained as previously described.^34^ The tissue was stored according to institutional ethics guidelines in a facility licensed by the Human Tissue Authority. Demographic and clinical characteristics are outlined in Supplementary Table 1. Sections were cleared in xylene and rehydrated in a graded ethanol series before being rinsed in distilled water and blocked for endogenous peroxidases with 0.3% H_2_O_2_ in methanol for 20 min. Slides were then blocked in Tris-buffered saline (TBS) with 0.3% Triton. Primary antibodies (Supplementary Table 2) were incubated in block solution overnight at 4°C and rinsed three times with TBS before the secondary antibodies (horseradish peroxidase anti-rat IgG *Sigma A9037*, HRP anti-mouse IgM *ThermoFisher 62-6820*) were added in TBS for 2 h at room temperature. Sections were again rinsed three times with TBS and then incubated in NovaRED peroxidase substrate (*Vector*) solution for 10 min. Slides were rinsed, dehydrated in a graded ethanol series and cleared in xylene before being mounted in xylene-based DPX media (*Sigma*). Modified Masson’s trichrome, omitting Weigert haematoxylin, was performed by the histology core (Department of Pathology, Cambridge U.K.).

### Mouse tissue immunohistochemistry

Spinal cord sections were cut using a cryostat (sagittal, 30 µm), mounted on slides and stored at −80°C. These were thawed, rinsed with phosphate-buffered saline (PBS) (+Ca^2+^/Mg^2+^) and blocked in PBS + 0.3% Triton X-100 + 5% BSA for 1 h at room temperature before primary antibodies were added (Supplementary Table 2) and incubated overnight at 4°C in blocking solution. For the mouse-on-mouse (CS56) antibody, a M.O.M blocking kit (*Abcam*) was used as per the manufacturer’s instructions to reduce endogenous IgG binding. Secondary antibodies (Alexa Fluor 568 goat anti-mouse IgM, Alexa Fluor 488 goat anti-rat IgG, Alexa Fluor 488 goat anti-rabbit IgG, Alexa Fluor 647 goat anti-rabbit IgG) were incubated in PBS for 2 h at room temperature (1:300), before final washing and mounting in mowiol (Sigma-Aldrich).

### Imaging

#### Human formalin-fixed spinal cords

For GFAP, CS56 and collagen IV quantification, five ×20 brightfield images were used per section. For vascular fibrosis quantification (collagen IV and trichrome), sections were imaged at ×20 and the ratio of the vessel wall to total lumen area was measured in FIJI. Trichrome analysis was performed by manual tracing and included 190 DCM group vessels and 144 from control tissues, while collagen IV analysis included 352 DCM and 339 control vessels. Interstitial fibrosis was measured as the blue signal from 10 ×40 brightfield images per sample using the channel deconvolution 2 plugin in FIJI. The anteroposterior compression ratio (APCR) was calculated by dividing the sagittal diameter by the transverse diameter.

#### Mouse

Sagittal sections were imaged using a Nikon Ti Eclipse confocal at ×20 either at the compression epicentre and at least 500 µm rostral or caudal the edge of visible compression. Vessel diameters (collagen IV, laminin, lectin) for at least five vessels in each image were measured by manual tracing in FIJI. GFAP and collagen I were quantified through total thresholded areas, while CS56 was quantified by particle size to reflect changes in distribution, rather than total area. All analyses were performed in FIJI.

### Bulk transcriptomic sequencing from the mouse DCM tissue

Total RNA was isolated from the snap-frozen PBS-perfused spinal cord tissue using the mirVana PARIS RNA and protein purification kit according to the manufacturer’s instructions. Bulk RNA-sequencing and library preparation were performed by the Schroeder Arthritis Center at Krembil Research Institute, Toronto. Read count abundances were generated from FASTQ files using *Salmon*. Protein-coding genes (*n* = 21 804) were isolated based on *GENCODE* biotype, and counts were imported into DESeq2 for normalization. Principal component analysis was performed using the *stats* package. Differential gene expression analysis was performed using DESeq2. Gene Ontology (GO) term enrichment analysis was performed using *clusterProfile*r (v4.12.6).

### Statistical analyses

For comparisons between two groups, *t*-tests or Mann–Whitney tests were performed as required. One- or two-way ANOVA or Kruskal–Wallis tests with multiple comparisons were used to compare means between three or more groups. Linear regression analyses were performed on continuous values for the cord area and compression ratio. These were performed using GraphPad Prism 10. Reported *n* values correspond to the number of individual mice or individual human subjects analysed.

## Results

### Transcriptomic signatures of DCM in mice

The degenerative SCI induced by this model was confirmed with haematoxylin and eosin (H&E) staining demonstrating cord compression and a lesion in the DCM group (Fig. 1B), along with significantly worsened mechanical allodynia (Fig. 1C) and forelimb stride length (Fig. 1D). Principal component analysis of the transcriptomic data at 4 weeks revealed two separate clusters corresponding with the DCM and sham groups (Fig. 1E). Among 21 804 protein-coding genes analysed, 2827 were significantly upregulated, and 2808 were downregulated (adjusted *P* < 0.05) in the DCM group relative to sham (Fig. 1F). GO overrepresentation analysis of genes upregulated in DCM revealed biological processes related to glial cell differentiation and morphogenesis. Enriched cellular compartments included the basement membrane, while molecular function terms included ECM constituent (Fig. 1G). In line with the principal components analysis and ontology overrepresentation analysis, broad differences in all subclasses of matrisome genes were evident (Fig. 1H).

### General characterization of human DCM

**Figure 2:**
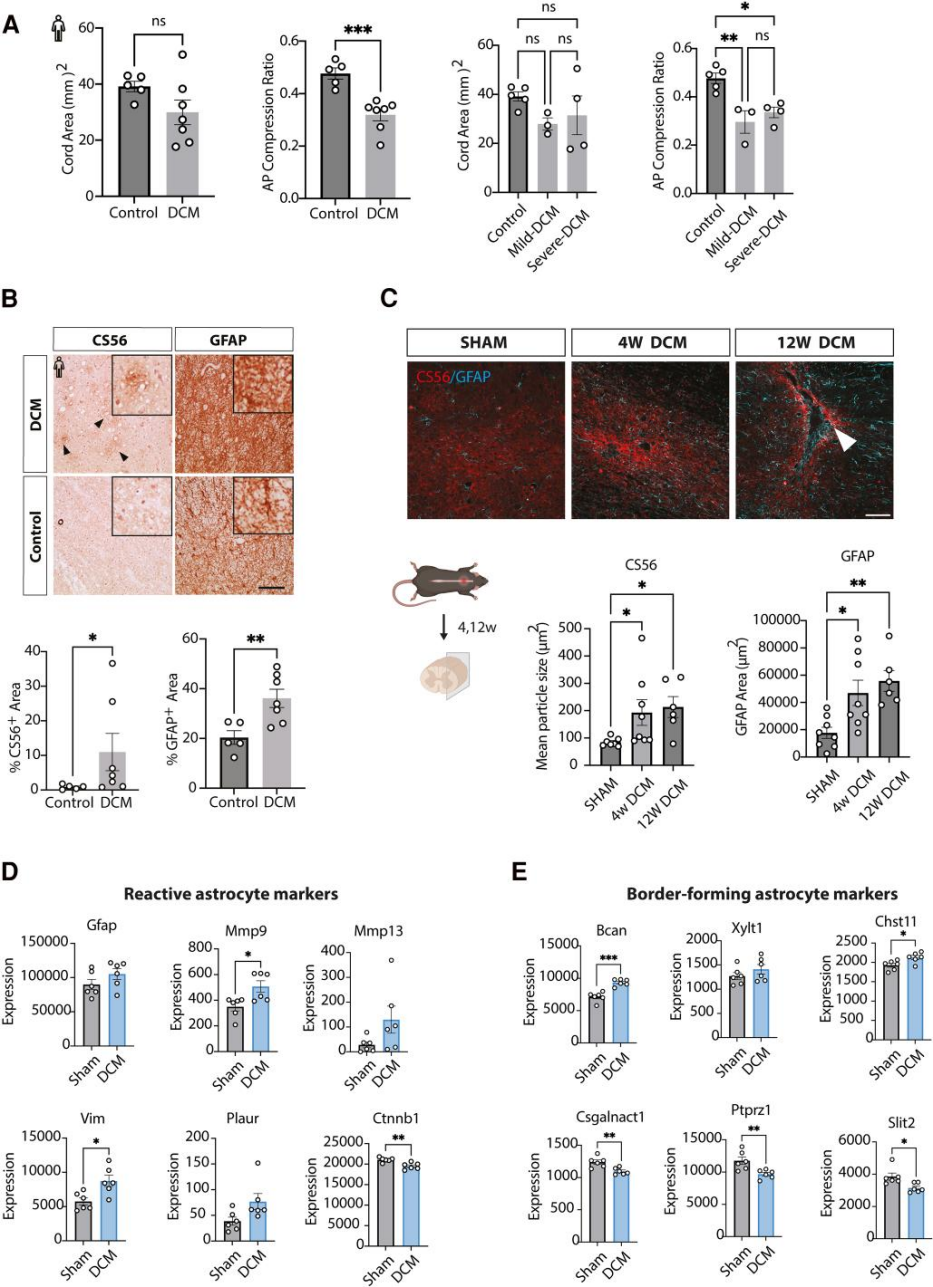
**Astrogliosis and CSPG deposition in human and mouse tissue.** (**A**) Comparison of the total cord area and APCR between DCM (*n* = 7 cases) and controls (*n* = 5) (area: *t*-test, *P* = 0.123, *t* = 1.684. APCR: *t*-test, *P* = 0.0006, *t* = 4.874) and between clinical severities of DCM (area ANOVA, F = 12.17, *P* = 0.0028 with Tukey’s correction for multiple comparisons). (**B**) Representative images and quantification of CSPGs and GFAP in the white matter of human spinal cords (GFAP: two-tailed *t*-test, *P* = 0.0096, *t* = 3.194 and CSPGs: Mann–Whitney test, *P* = 0.048, U = 5, both *n* = 7 DCM cases, *n* = 5 control cases). Scale bar = 100 µm. Black arrows indicate regions of CSPG deposition. (**C**) Experiment schematic created in BioRender https://BioRender.com/tolldo2. Representative images and quantification of CSPGs and GFAP at the compression epicentre in mouse spinal cord tissue from sham (*n* = 7 mice), 4w DCM (*n* = 8 mice) and 12w DCM (*n* = 6 mice) (GFAP, one-way ANOVA, *P* = 0.0043, F = 7.343; CSPG, Kruskal–Wallis test, *P* = 0.013, KW statistic 8.68, scale bar = 100 µm, white arrow denotes a lesion border at 12 weeks of DCM). (**D**) mRNA expression levels of reactive astrocyte markers in mouse samples from bulk RNA-sequencing (*t*-tests or Mann–Whitney tests, *n* = 6 mice per group). (**E**) mRNA expression levels of border-forming astrocyte markers in mouse samples (*t*-tests or Mann–Whitney tests, *n* = 6 mice per group). Data points shown represent the mean value of individual animals or individual human cases ± standard error of the mean. Significance levels are shown as *P < 0.05, **P < 0.01, ***P < 0.001 and ***P < 0.0001 for all comparisons.

Seven patients and five controls were analysed. The clinical severity of DCM was either mild or severe and is summarized in Supplementary Table 1. To assess compression of the spinal cord, we calculated the APCR. APCR was significantly lower in the DCM group relative to controls. However, there were no significant differences between the clinically severe and mild DCM tissue (Fig. 2A). The overall cord area was not significantly lower in DCM (Fig. 2A).

### Interstitial and vascular fibrosis

**Figure 3:**
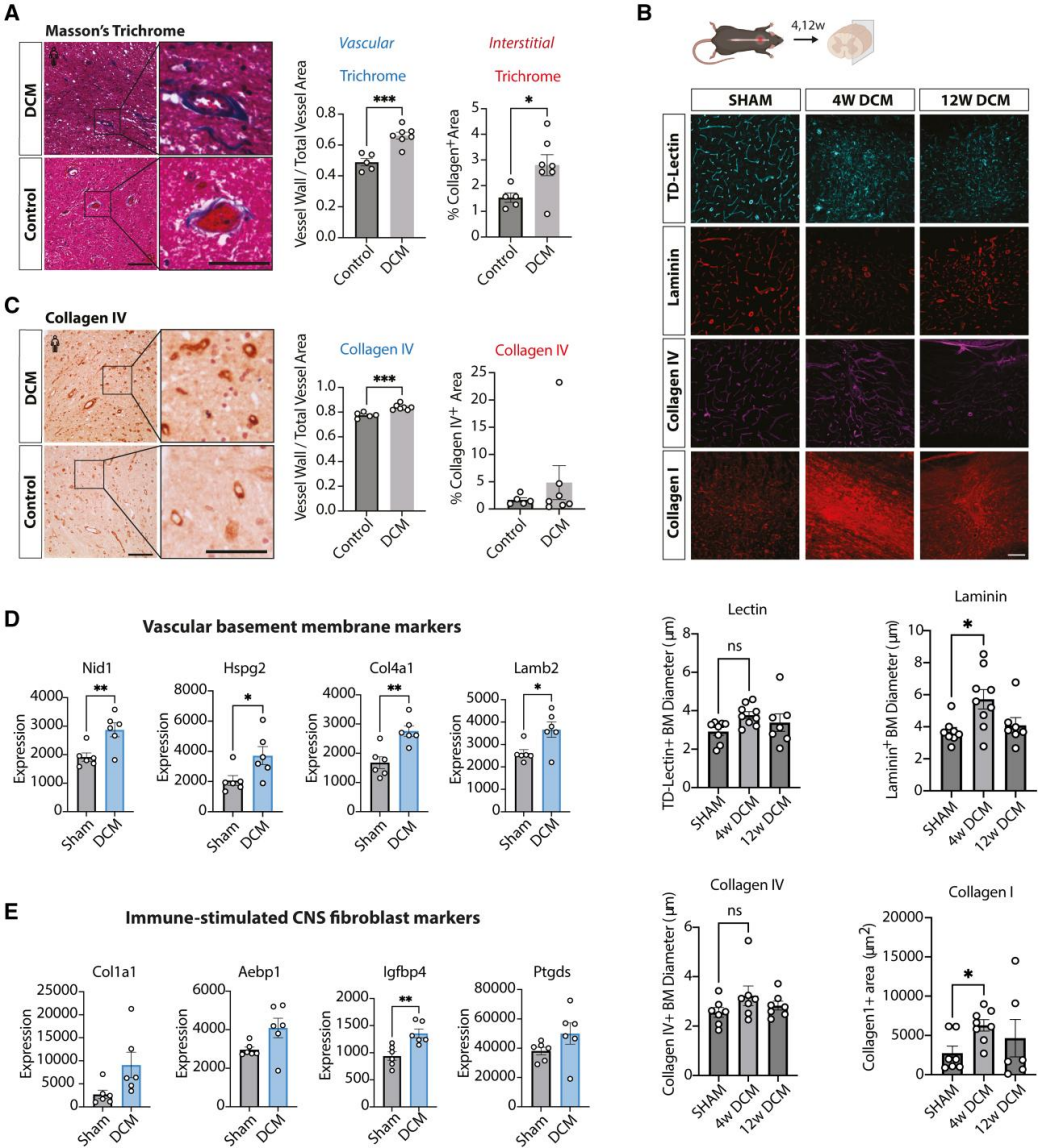
**Interstitial and vascular fibrosis in human and mouse tissue.** (**A**) Masson’s Trichrome staining of vascular and interstitial collagen fibrils in control cases (*n* = 5) and DCM cases (*n* = 7) (vascular fibrosis, *t*-test, *P* = 0.0004, *t* = 5.133; interstitial fibrosis, Welch’s *t*-test, *P* = −0.0223, *t* = 2.816). Scale bar = 100 µm. (**B**) Experiment schematic created in BioRender https://BioRender.com/wrfl28u. Representative images and quantification of interstitial collagen I, vascular collagen IV, vascular TD-Lectin and vascular laminin in 4- and 12-week mouse spinal cord sagittal sections. Scale bar = 100 µm and 50 µm in magnified view, two-way ANOVA with multiple comparisons, details in Supplementary Table 3, *n* = at least 5 mice per group. (**C**) Representative images and quantification of vascular and interstitial collagen IV in human tissue (two-tailed *t*-test, *n* = 7 DCM cases, *n* = 5 control cases). Scale bar = 100 µm and 50 µm in magnified view. (**D**) mRNA expression levels of vascular basement membrane markers in DCM (*t*-tests or Mann–Whitney tests, *n* = 6 mice per group). (**E**) mRNA expression levels of activated fibroblast markers in DCM (*t*-tests or Mann–Whitney tests, *n* = 6 mice per group). Data points shown represent the mean value of individual animals or individual human cases ± standard error of the mean. Significance levels are shown as *P < 0.05, **P < 0.01, ***P < 0.001 and ***P < 0.0001 for all comparisons.

**Figure 4:**
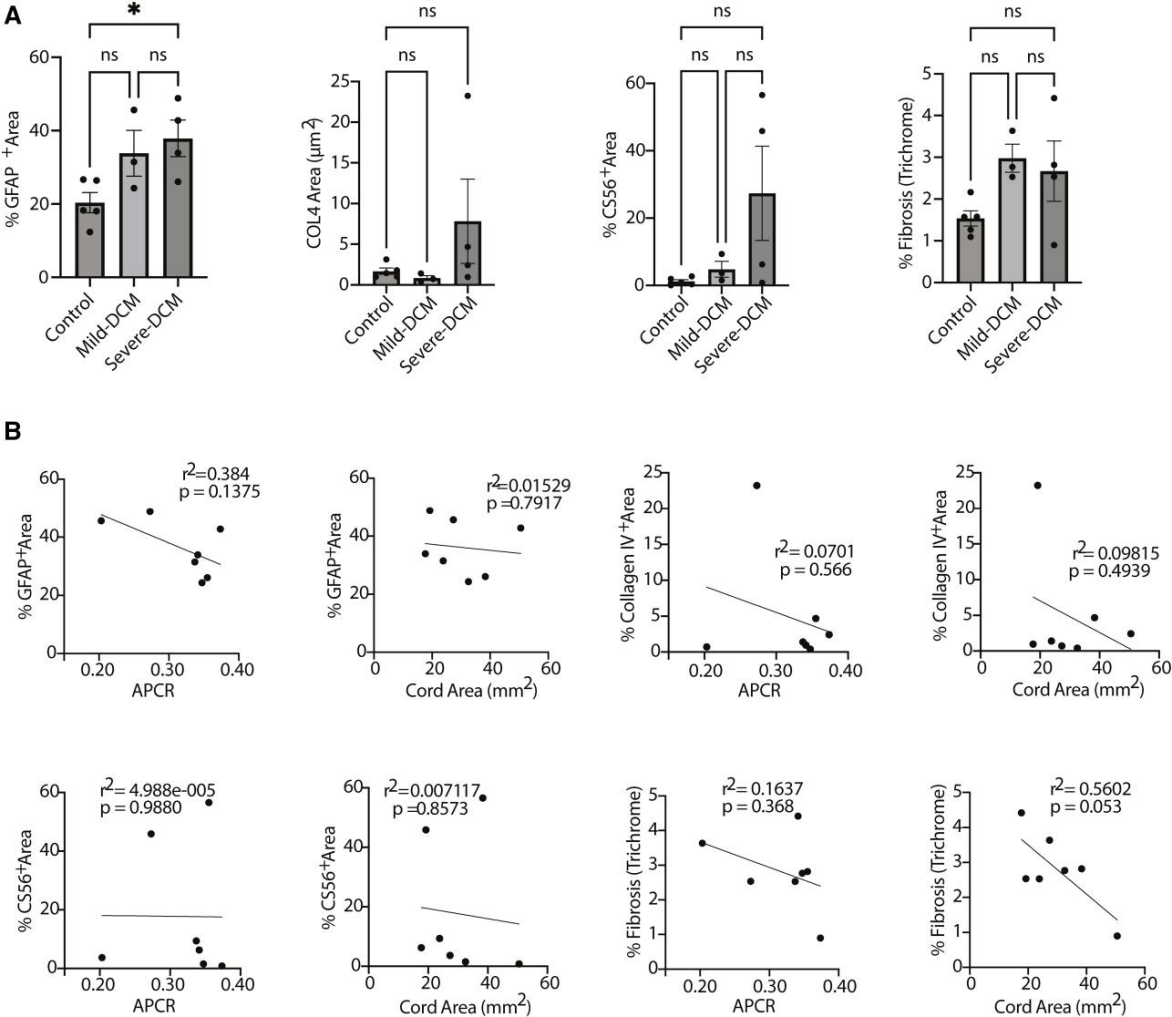
**Associations between gliosis, fibrosis and extent of cord compression in untreated post-mortem human DCM samples.** (**A**) Comparison of CS56, GFAP, collagen IV and fibrotic area between clinical severities (one-way ANOVA, details in Supplementary Table 3, *n* = 7 DCM cases, *n* = 5 control cases). (**B**) Correlations between CS56, GFAP, collagen IV or fibrotic area and APCR or cord area (linear regression, *n* = 7 DCM cases, *n* = 5 control cases). Data points shown represent individual human cases ± standard error of the mean. Significance levels are shown as *P < 0.05, **P < 0.01 and ***P < 0.001.

Severe CNS injury is generally marked by fibrous collagen I at the epicentre, with CSPG deposition, collagen IV and laminin largely at the lesion borders. Here, in human samples, Masson’s trichrome staining showed elevated deposition of collagen in DCM, suggestive of fibrosis (Fig. 3A). The trichrome-positive area was not significantly correlated with APCR or clinical severity (Fig. 4A and B). Interstitial collagen IV was not significantly higher in the human DCM group, and levels were not correlated with APCR or clinical severity (Figs 3C and 4A and B). With respect to vascular fibrosis, in the post-mortem DCM samples, collagen IV^+^ and Masson-stained vessel wall area ratio was significantly increased (Fig. 3A and C).

In the mouse model, the immune-stimulated CNS fibroblast genes *Col1a1* and *Aebp1* were not significantly elevated in the DCM group, although *Igfbp4* was statistically higher than sham (Fig. 3E). Meanwhile, the laminin^+^, but not lectin^+^ and collagen IV^+^ basement membrane diameters, was significantly increased at 4 weeks of DCM (Fig. 3B). Collagen I areas were increased at 4 but not 12 weeks of DCM. At the transcriptional level, vascular ECM genes collagen IV (*Col4a1*), laminin (*Lamb2*), *Nid1* and *Hspg2* were significantly upregulated at 4 weeks of DCM (Fig. 3D).

### CSPG deposition and remodelling

Common markers of astrogliosis and astrocyte matrix deposition include GFAP and the CSPG marker CS56, respectively. Here, astrogliosis and CSPG deposition were measured by immunohistochemistry in human post-mortem tissue. In the DCM group, GFAP and CS56 expression was significantly increased relative to healthy controls (*P*  *<* 0.05, Mann–Whitney and unpaired *t*-tests) (Fig. 2B). Moreover, mean APCR and clinical severity scores did not correlate with levels of CS56- or GFAP-positive area (Fig. 4A and B).

In the mouse DCM model, immunohistochemistry revealed significant upregulation of CS56 and GFAP in the epicentre, with diffuse CSPG expression at 4 weeks transforming into a defined lesion border by 12 weeks. In shams, CS56 immunoreactivity was limited to perineuronal nets (Fig. 2C). At the transcriptional level, there was upregulation of reactive astrocyte markers *Vimentin* and *Mmp9,* although *Ctnnb1* was downregulated. The border-forming astrocyte ECM genes *Bcan* and *Chst11* were elevated in DCM, while *Csgalnact1*, *Slit2* and phosphacan *(Ptprz1)* were downregulated (Fig. 2D and E).

## Discussion

### Transcriptomic analysis of the mouse DCM spinal cord tissue

To characterize matrix changes in degenerative myelopathy, we first examined bulk transcriptional profiles in mice. GO enrichment analysis revealed differential regulation of several pathways associated with CNS injury and matrix remodelling at 4 weeks. Future studies could provide a longitudinal characterization of chronic spinal cord compression through transcriptomic analyses at an expanded set of acute and chronic timepoints. We next sought to characterize the extent of ECM deposition in DCM at the protein level, based on known cellular responses in traumatic SCI.

### Border-forming astrogliosis is evident in an animal model, but not human samples

Gliosis has been referenced in histopathological studies of DCM,^29^ but quantification of specific protein-level markers has not been reported. Notably, although CSPG deposition is known to be present in traumatic SCI,^12,13^ it is unknown whether CSPG deposition occurs with chronic spinal cord compression and myelopathy. In the untreated post-mortem DCM samples analysed here, CSPG deposition appeared patchy and scattered without the formation of dense, border-like structures. Another lesion border-associated matrix protein, collagen IV,^35^ was neither significantly upregulated in the untreated DCM tissue nor observed as isolated regions of immunoreactivity, unlike the CSPGs. It is likely that both astrocytes^35,36^ and fibroblasts^37^ contribute collagen IV in spinal cord lesions. Nevertheless, the lack of any border-associated collagen IV in these samples suggests that DCM primarily induces classic reactive astrogliosis, with minimal lesion border formation in human samples. Therefore, the reactive astroglial phenotype in DCM differs from traumatic or penetrating CNS injuries. DCM results from prolonged mechanical stress, ischaemia and hypoxia at lower intensities and over a longer duration than traumatic SCI. Given the relatively lower degree of tissue disruption, glial border formation may play a limited role in preventing lesion spread. This could be attributed to predominantly apoptotic^38^ rather than necrotic cell death, as low levels of necrosis would not necessitate the same extent of lesion containment observed in SCI.

In the mouse model of DCM, however, focal reactive astrocyte lesions were more prominent at 12 weeks and characterized by overlapping areas of dense CS56 and GFAP expression resembling lesion borders. CS56 and GFAP areas were significantly elevated compared to sham animals at both timepoints. In the sham tissue, CSPG expression was restricted to perineuronal structures. Despite our attempt to match the timing and progression of human DCM in the mouse model, the timing of compression onset may still have been more acute and hence severe than the human cases here. Moreover, the lack of an evident astrocyte border in the human samples may be due to axial sectioning. The mouse model we used is most representative of ossification of the ligamentum flavum, which is one recognized aetiology of DCM. There may be histological differences between aetiologies of DCM (e.g. spondylotic myelopathy versus OPLL versus ossification of the ligamentum flavum), and therefore, the mouse model histology may not perfectly reflect that of the human samples. In prior experimental models of DCM, the glial scar tissue was quantified by H&E staining and found to be elevated relative to sham rostral and caudal to the injury epicentre, though molecular markers were not assessed.^39^ Similarly, another study in rats quantified the glial scar tissue using H&E and reported a significant increase at the epicentre.^20^

Our findings provide molecular confirmation of these reports in animal models, suggesting that astrogliosis and CSPG deposition occur in DCM but follow a distinct pattern from the lesion border-related deposition seen in traumatic SCI.

### Fibrosis-associated interstitial matrix deposition

Chronic fibrosis in CNS lesions is characterized by the deposition of type I collagen.^40,41^ Here, trichrome staining demonstrated that interstitial collagen was elevated in DCM. This suggests that DCM pathophysiology shares molecular similarities with traumatic injury. However, the collagen area was not significantly correlated with the extent of compression or clinical severity, and interstitial collagen IV was not elevated in DCM. In the mouse model, fibrotic scar-related collagen deposition was more pronounced and distinct interstitial deposition of collagen I was observed at both 4 and 12 weeks. Correspondingly, mRNA expression of several fibroblast activation genes was elevated in DCM.

### Vascular fibrosis

The thickened vessel walls observed in the post-mortem human samples likely result, at least in part, from endothelial damage and the subsequent local activation of vascular fibroblasts or pericytes.^42^ Distinguishing the relative contributions of ischaemic inflammatory insults versus shear-stress-mediated endothelial damage remains challenging. Notably, collagen I and II—but not collagen IV—have been associated with cerebral small vessel disease and white matter lesions.^43^ Significant differences in vessel wall thickness were observed between 4-week DCM and sham mice, and these differences were no longer evident at 12 weeks. This may be due in part to vascular remodelling and sprouting that is known to occur in ischaemic conditions.^44^ Prior rodent studies support the notion of BSCB disruption in DCM. In a rat model, Evans blue dye intensity was increased, with effects extending beyond the injury epicentre.^20^ Notably, matrix metalloproteinases, key enzymes involved in BSCB remodelling, have also previously been shown to be increased in DCM.^21^ Recent findings from contusion and transection SCI^37^ suggest that vascular fibroblasts contribute to outer lesion border formation by producing laminin and collagen IV, whereas meningeal-derived fibroblasts deposit collagen I and fibronectin in the lesion core. Although pericytes and vascular smooth muscle cells were not found to contribute to fibrotic scar formation, they may play a role in local basement membrane thickening.^37^ Pericytes, fibroblasts, astrocytes and endothelial cells all contribute various isoforms of collagen and laminin to the vascular basal lamina, along with cross-linking molecules like nidogen.^45^ Future single-cell transcriptomic analyses at chronic timepoints (e.g. 12 weeks) could more definitively identify which cells contribute to vascular fibrosis in DCM.

### Histopathological correlates with morphology in human tissues

Some human histological studies suggest that pathological changes become more severe as compression increases,^17,46,47^ while others have reported that the transverse area and shape^18,48^ correlates with degree of pathological change more than compression. Here, we assessed both the APCR and the area. The compression ratio was significantly lower in the DCM group relative to controls. However, there were no significant differences between clinically severe and mild DCM tissue. Moreover, the overall cord area was not significantly lower in DCM relative to controls. The morphological results should be interpreted with caution because of potential shrinkage artefacts with formalin fixation. These observations are further limited by sample size. The correlations between clinical severity and histology in our cohort are limited by the presence of other neurological pathology (e.g. cerebral infarction, dementia) that were not known to involve the spinal cord but may have affected the original severity classification due to overlapping symptoms.

In conclusion, our findings demonstrate that chronic compressive SCI is associated with ECM changes that partially overlap with, yet are distinct from, those seen in acute traumatic injury. We demonstrate that astrogliosis-associated CSPG deposition does occur in DCM, along with vascular and interstitial fibrosis. These findings highlight potential targets for therapies aimed at enhancing neuroplasticity and modulating inflammation.

## Supplementary Material

fcag239_Supplementary_Data

## Data Availability

Transcriptomic data generated in this study are available as raw counts. Code associated with data analysis is available at https://github.com/noahdavidpoulin/ECM-in-DCM.
