# Supplementary material for: Extracellular matrix remodelling in degenerative cervical myelopathy
Source: Brain Commun. 2026 Jul 23;8(4):fcag239. doi: 10.1093/braincomms/fcag239 (PMC13392463; doi:10.1093/braincomms/fcag239)
Supplement: fcag239_Supplementary_Data [file fcag239_supplementary_data.docx]

# Supplementary Materials: Extracellular matrix remodelling in degenerative cervical myelopathy

**Supplementary Table 1: Patient characteristics**

| **Case** | **Sex** | **Age** | **Duration of illness (years)** | **No. of symptom groups** | **Severity** | **Other neurological pathology** |
| --- | --- | --- | --- | --- | --- | --- |
| 1 | M | 80 | Unknown | 1 | Mild | Cerebral infarct |
| 2 | M | 68 | 1.3 | 2 | Mild | Nil |
| 3 | M | 63 | 10 | 3 | Mild | Dementia |
| 4 | M | 59 | 8 | 4 | Severe | Cerebral infarct |
| 5 | M | 72 | 2.8 | 4 | Severe | Nil |
| 6 | F | 72 | 4 | 5 | Severe | Nil |
| 7 | M | 61 | 2 | 6 | Severe | Brain stem neuroma |
| **Controls** |  |  |  |  |  |  |
| 1 | F | 73 |  |  |  | Nil |
| 2 | F | 83 |  |  |  | Nil |
| 3 | F | 68 |  |  |  | Nil |
| 4 | M | 65 |  |  |  | Malignant lymphoma in cerebrum |
| 5 | M | 51 |  |  |  | Nil |

**Supplementary Table 2: Antibodies**

| **Antigen** | **Species** | **Manufacturer & Cat #** | **Concentration (application)** |
| --- | --- | --- | --- |
| CS56 | Mouse | Sigma C8035 | 1:200 (IHC) 1:100 (IHC-P) |
| GFAP | Rat | ThermoFisher 13-0300 | 1:300 (IHC) 1:200 (IHC-P) |
| Collagen IV | Rabbit | Abcam ab6586 | 1:300 (IHC) 1:300 (IHC-P) |
| Collagen I | Rabbit | Abcam ab34710 | 1:300 (IHC), 1:500 (WB) |
| Laminin | Rabbit | Abcam ab11575 | 1:300 (IHC) |
| TD-Lectin | Alexa-649 Conjugated | Vector Lab 1178 | 1:300 (IHC) |

**Supplementary Table 3: ANOVA statistical testing details for Figures 1, 3, and 4**

| **Figure/Panel** | **Factors** | **F-statistic** | **P-value** | **Significant Comparisons** |
| --- | --- | --- | --- | --- |
| **Fig 1 / C** | Interaction: Time x Group | 8.346 | P<0.0001 |  |
| **Fig 1 / D** | DCM vs Sham (Group) | 5.744 | 0.0232 |  |
| **Fig 2 / A** | APCR | 12.17 | 0.0028 | Severe-DCM vs control (adjusted p=0.0111)  Mild-DCM vs control (adjusted p = 0.0041) |
| **Fig 3 / B** | Laminin | 5.086 | 0.0158 | Sham vs 4W DCM (adjusted p = 0.0128 |
| **Fig 3 / B** | Collagen I (Welch’s) | W = 4.478 | 0.0414 | Sham vs 4W DCM (adjusted p = 0.0183) |
| **Fig 4 / A** | GFAP | 4.972 | 0.0351 | Control vs Severe-DCM (adjusted p = 0.0361) |
